# Autophagy controls centrosome number

**DOI:** 10.18632/oncotarget.15362

**Published:** 2017-02-15

**Authors:** Shinya Honda, Shigeomi Shimizu

**Affiliations:** Department of Pathological Cell Biology, Medical Research Institute, Tokyo Medical and Dental University, Bunkyo-ku, Tokyo, Japan

**Keywords:** Autophagy, centrosome, Cep63

It is believed that the number of centrosomes in a cell is tightly regulated by the degradation of centrosomal proteins via the ubiquitin–proteasome protein degradation system. However, we recently demonstrated that autophagy also participates in the regulation of centrosome number.

The centrosome is the major component of the microtubule organizing center of mammalian cells, and plays an essential role in chromosome segregation during cell division. Centrosome number is tightly regulated during the cell cycle: G1 cells have one mature centrosome containing a pair of centrioles. Centriole replication occurs during the S phase, and each centriole duplicates to produce two daughter centrioles, to generate two centrosomes in the G2–M phase. If cells have more than three centrosomes during metaphase, this causes chromosome missegregation and genomic instability. Most such cells eventually die owing to their abnormal number of chromosomes, but the cells that remain acquire malignant potential. Centrosomes also play a role in ciliogenesis. Upon cell cycle exit, centrosomes migrate to the cell surface and form basal bodies, which are required for nucleation of ciliary axonemes [1]. Thus, an abnormal centrosome number also disrupts cilia-dependent cellular events.

We recently found that autophagy plays a role in maintaining the proper number of centrosomes [2]. Our first observation was the presence of multiple centrosomes in cells lacking Atg5, which is a key molecule of autophagy, using electron microscopy. This finding was readily confirmed by γ-tubulin immunostaining (the most reliable marker of centrosome). Consistently, cells lacking other autophagy molecules, such as Ulk1, Beclin1, and Atg7, have multiple centrosomes, indicating the essential role of autophagy in the maintenance of proper centrosome number. We first hypothesized that autophagy degrades excess mature centrosomes. However, we found no such evidence. Instead, we observed the following characteristic features of centrosomal protein 63 kDa (Cep63), which is a molecule that functions in the initial step of centriole duplication: (1) most Cep63 dots were enclosed in autolysosomes in wild-type (WT) cells upon the inhibition of autolysosomal degradation using lysosomal inhibitors, (2) the number of Cep63 dots was increased time-dependently in WT cells by the administration of autophagy inhibitors, (3) the number of Cep63 dots in autophagy-deficient cells was more than that in normal cells, and (4) silencing Cep63 decreased the number of centrosomes in autophagy-deficient cells. From these findings, we concluded that Cep63 dots are the direct substrates of autophagy and multiple centrosomes are generated from the excess Cep63 dots in autophagy-deficient cells.

**Figure 1:**
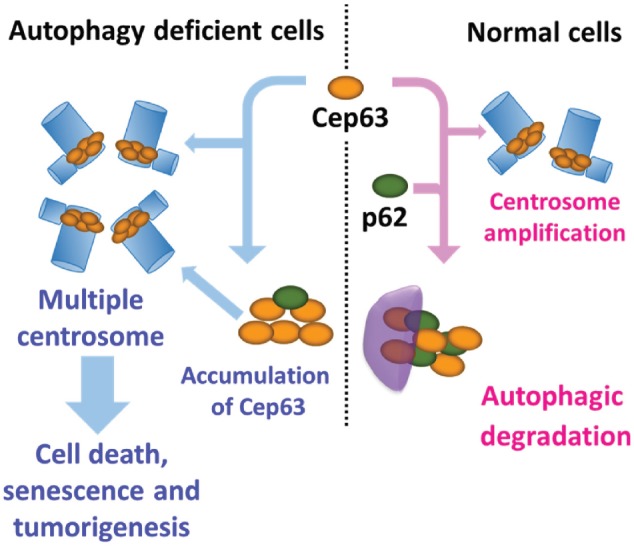
Regulation of centrosome number by autophagy In normal cells, newly synthesized Cep63 dots are degraded by p62-dependent selective autophagy. In autophagy-deficient cells, Cep63 dots accumulate and generate an excess number of centrosomes.

Consistent with these findings, in autophagy-deficient cells, most Cep63 dots directly bound to p62, an adaptor or cargo receptor for autophagic degradation. These p62-associated Cep63 dots were not become mature centrosomes, whereas some Cep63 dots become mature centrosomes by their interaction with Cep152, instead of p62. Cep152 is the first protein to interact with Cep63 in the initial step of centriole duplication (Figure 1).

Our data indicated that Cep63 dots are continuously generated and rapidly degraded by autophagy. What is the biological role of this continuous generation of Cep63 dots? One possible role is a contribution in multiciliogenesis. Several types of terminally differentiated cells, including cells on the surface of the brain ventricles, oviduct, and trachea, have multicilia. To generate multicilia, cells require the amplification of many centrioles in a cell cycle-independent manner. For this purpose, Cep63 dots need to be generated continuously. This idea is supported by a recent study indicating the regulation of multiciliogenesis by Cep63 and Deup1, a paralogue of Cep63 [3]. In terminally differentiated cells, cells will use the continuously generated Cep63 for ciliogenesis, probably coupled with a reduction in autophagic activity.

Taken together, the number of centrosomes is tightly regulated by both the ubiquitin–proteasome system as well as autophagy. Both machineries degrade various molecules and have different cell cycle dependency. The ubiquitin–proteasome system mainly functions in a cell cycle-dependent manner, *i.e.*, SCF ubiquitinates Plk4 in a cell cycle-dependent manner [4, 5]. In contrast, autophagy degrades Cep63 in a cell cycle-independent manner. Therefore, these two systems share roles to maintain the proper number of centrosomes in cells.
